# Supplementary material for: Modeling glioblastoma heterogeneity as a dynamic network of cell states
Source: Mol Syst Biol. 2021 Sep 16;17(9):e10105. doi: 10.15252/msb.202010105 (PMC8444284; doi:10.15252/msb.202010105)
Supplement: Supplementary file 5 — Source Data for Figure 3 [file MSB-17-e10105-s001.zip › Figure3A_sourcedata/GSEA_3065/hallmarks_state2.GseaPreranked.1623416269049/HALLMARK_IL6_JAK_STAT3_SIGNALING.html]

Details for gene set HALLMARK\_IL6\_JAK\_STAT3\_SIGNALING[GSEA]

|  || Dataset | state2 |
| Phenotype | NoPhenotypeAvailable |
| Upregulated in class | na\_neg |
| GeneSet | HALLMARK\_IL6\_JAK\_STAT3\_SIGNALING |
| Enrichment Score (ES) | -0.6231842 |
| Normalized Enrichment Score (NES) | -1.9232193 |
| Nominal p-value | 0.0 |
| FDR q-value | 2.1527779E-4 |
| FWER p-Value | 0.001 |
Table: GSEA Results Summary

  

Fig 1: Enrichment plot: HALLMARK\_IL6\_JAK\_STAT3\_SIGNALING      
 Profile of the Running ES Score & Positions of GeneSet Members on the Rank Ordered List

  

| PROBE | GENE SYMBOL | GENE\_TITLE | RANK IN GENE LIST | RANK METRIC SCORE | RUNNING ES | CORE ENRICHMENT || 1 | IFNGR2 |  |  | 1538 | 0.085 | -0.1350 | No |
| 2 | PTPN11 |  |  | 1665 | 0.079 | -0.1218 | No |
| 3 | JUN |  |  | 1805 | 0.072 | -0.1123 | No |
| 4 | STAT1 |  |  | 2336 | 0.053 | -0.1509 | No |
| 5 | HAX1 |  |  | 2593 | 0.045 | -0.1630 | No |
| 6 | CBL |  |  | 2642 | 0.043 | -0.1536 | No |
| 7 | IL17RB |  |  | 2712 | 0.041 | -0.1470 | No |
| 8 | TYK2 |  |  | 3547 | 0.023 | -0.2279 | No |
| 9 | MYD88 |  |  | 3818 | 0.019 | -0.2504 | No |
| 10 | STAM2 |  |  | 3826 | 0.018 | -0.2449 | No |
| 11 | GRB2 |  |  | 3991 | 0.016 | -0.2571 | No |
| 12 | BAK1 |  |  | 4847 | 0.002 | -0.3474 | No |
| 13 | PTPN1 |  |  | 5037 | -0.001 | -0.3673 | No |
| 14 | IL17RA |  |  | 5379 | -0.006 | -0.4014 | No |
| 15 | PTPN2 |  |  | 5582 | -0.010 | -0.4196 | No |
| 16 | SOCS3 |  |  | 6224 | -0.020 | -0.4809 | No |
| 17 | IRF1 |  |  | 7563 | -0.052 | -0.6056 | Yes |
| 18 | IFNAR1 |  |  | 7683 | -0.056 | -0.5995 | Yes |
| 19 | IL13RA1 |  |  | 7762 | -0.058 | -0.5881 | Yes |
| 20 | OSMR |  |  | 7864 | -0.062 | -0.5780 | Yes |
| 21 | A2M |  |  | 7912 | -0.063 | -0.5617 | Yes |
| 22 | TNFRSF12A |  |  | 7931 | -0.064 | -0.5420 | Yes |
| 23 | IFNGR1 |  |  | 7945 | -0.065 | -0.5216 | Yes |
| 24 | PDGFC |  |  | 8287 | -0.082 | -0.5303 | Yes |
| 25 | TGFB1 |  |  | 8497 | -0.095 | -0.5204 | Yes |
| 26 | CSF1 |  |  | 8560 | -0.101 | -0.4931 | Yes |
| 27 | TNFRSF1A |  |  | 8962 | -0.143 | -0.4875 | Yes |
| 28 | HMOX1 |  |  | 9084 | -0.163 | -0.4456 | Yes |
| 29 | IL6ST |  |  | 9090 | -0.164 | -0.3909 | Yes |
| 30 | STAT2 |  |  | 9134 | -0.174 | -0.3369 | Yes |
| 31 | TNFRSF21 |  |  | 9223 | -0.202 | -0.2783 | Yes |
| 32 | CD9 |  |  | 9324 | -0.248 | -0.2054 | Yes |
| 33 | STAT3 |  |  | 9325 | -0.249 | -0.1215 | Yes |
| 34 | CD44 |  |  | 9407 | -0.397 | 0.0035 | Yes |
Table: GSEA details [plain text format]

  

Fig 2: HALLMARK\_IL6\_JAK\_STAT3\_SIGNALING: Random ES distribution      
 Gene set null distribution of ES for **HALLMARK\_IL6\_JAK\_STAT3\_SIGNALING**
